# Rapid optimization of enzyme mixtures for deconstruction of diverse pretreatment/biomass feedstock combinations

**DOI:** 10.1186/1754-6834-3-22

**Published:** 2010-10-12

**Authors:** Goutami Banerjee, Suzana Car, John S Scott-Craig, Melissa S Borrusch, Jonathan D Walton

**Affiliations:** 1 Department of Energy Great Lakes Bioenergy Research Center and Department of Energy Plant Research Laboratory, Michigan State University, East Lansing MI 48824, USA

## Abstract

**Background:**

Enzymes for plant cell wall deconstruction are a major cost in the production of ethanol from lignocellulosic biomass. The goal of this research was to develop optimized synthetic mixtures of enzymes for multiple pretreatment/substrate combinations using our high-throughput biomass digestion platform, GENPLAT, which combines robotic liquid handling, statistical experimental design and automated Glc and Xyl assays. Proportions of six core fungal enzymes (CBH1, CBH2, EG1, β-glucosidase, a GH10 endo-β1,4-xylanase, and β-xylosidase) were optimized at a fixed enzyme loading of 15 mg/g glucan for release of Glc and Xyl from all combinations of five biomass feedstocks (corn stover, switchgrass, *Miscanthus*, dried distillers' grains plus solubles [DDGS] and poplar) subjected to three alkaline pretreatments (AFEX, dilute base [0.25% NaOH] and alkaline peroxide [AP]). A 16-component mixture comprising the core set plus 10 accessory enzymes was optimized for three pretreatment/substrate combinations. Results were compared to the performance of two commercial enzymes (Accellerase 1000 and Spezyme CP) at the same protein loadings.

**Results:**

When analyzed with GENPLAT, corn stover gave the highest yields of Glc with commercial enzymes and with the core set with all pretreatments, whereas corn stover, switchgrass and *Miscanthus *gave comparable Xyl yields. With commercial enzymes and with the core set, yields of Glc and Xyl were highest for grass stovers pretreated by AP compared to AFEX or dilute base. Corn stover, switchgrass and DDGS pretreated with AFEX and digested with the core set required a higher proportion of endo-β1,4-xylanase (EX3) and a lower proportion of endo-β1,4-glucanase (EG1) compared to the same materials pretreated with dilute base or AP. An optimized enzyme mixture containing 16 components (by addition of α-glucuronidase, a GH11 endoxylanase [EX2], Cel5A, Cel61A, Cip1, Cip2, β-mannanase, amyloglucosidase, α-arabinosidase, and Cel12A to the core set) was determined for AFEX-pretreated corn stover, DDGS, and AP-pretreated corn stover. The optimized mixture for AP-corn stover contained more exo-β1,4-glucanase (i.e., the sum of CBH1 + CBH2) and less endo-β1,4-glucanase (EG1 + Cel5A) than the optimal mixture for AFEX-corn stover. Amyloglucosidase and β-mannanase were the two most important enzymes for release of Glc from DDGS but were not required (i.e., 0% optimum) for corn stover subjected to AP or AFEX. As a function of enzyme loading over the range 0 to 30 mg/g glucan, Glc release from AP-corn stover reached a plateau of 60-70% Glc yield at a lower enzyme loading (5-10 mg/g glucan) than AFEX-corn stover. Accellerase 1000 was superior to Spezyme CP, the core set or the 16-component mixture for Glc yield at 12 h, but the 16-component set was as effective as the commercial enzyme mixtures at 48 h.

**Conclusion:**

The results in this paper demonstrate that GENPLAT can be used to rapidly produce enzyme cocktails for specific pretreatment/biomass combinations. Pretreatment conditions and feedstock source both influence the Glc and Xyl yields as well as optimal enzyme proportions. It is predicted that it will be possible to improve synthetic enzyme mixtures further by the addition of additional accessory enzymes.

## Background

The cost of enzymes for the release of fermentable sugars from plant cell wall polysaccharides remains one of the major hurdles to the development of an economically viable cellulosic ethanol industry [1,2]. Currently available enzyme mixtures are complex and only partially defined mixtures of more than 80 proteins [3]. A better understanding of which enzymes, and their proportions, are important for lignocellulosic degradation could eventually lead to the rational design of more efficient, and hence less expensive, enzyme mixtures. As an approach to addressing this problem, a high-throughput analysis platform, called GENPLAT, has been developed. GENPLAT utilizes individual purified enzymes, statistical experimental design, robotic pipetting of slurries and enzymes, and automated colorimetric determination of released sugars [4,5]. With GENPLAT, it is possible to optimize mixtures of pure enzymes for the release of Glc and Xyl from different substrates.

Several laboratories have shown that it is possible to construct multicomponent mixtures of pure endo- and exocellulases, endoxylanases and debranching enzymes that equal (for Glc) or surpass (for Xyl) commercial preparations [4-8]. Our earlier studies focused on a single pretreatment/substrate, namely, ammonia fiber expansion (AFEX)-treated corn stover. It would be desirable, however, for ethanol producers to be able to utilize a variety of lignocellulosic feedstocks, including different grass stovers (e.g., sorghum, switchgrass or *Miscanthus*) as well as other biomass materials such as corn cobs, dried distillers' grains (DDG) or mixed native prairie [9,10]. Because different feedstocks respond differently to different pretreatment conditions (e.g., steam, hot water, ionic liquids, dilute acid, AFEX, or alkaline peroxide), there are a large number of possible pretreatment/substrate combinations. The lignocellulosic industry of the future will likely use many different pretreatment/biomass combinations, and it will therefore be necessary to have enzyme mixtures that can handle all of these different pretreatment/biomass combinations. Currently available commercial enzyme preparations are limited in number and composition and have generally been optimized for acid-pretreated stover from corn and other grasses. They are therefore unlikely to be well adapted for the spectrum of pretreatment/biomass combinations that exist now or that may emerge in the future.

One solution to this problem is to design enzyme mixtures for specific applications. Given a sufficiently abundant source of individual pure enzymes, cocktails could be designed for specific pretreatment/biomass combinations, plant species, cultivars or even individual batches of biomass as they are delivered to the processing site. To do so, there must be a method to rapidly optimize custom cocktails. We here show that GENPLAT can be used to construct multicomponent synthetic enzyme cocktails for release of Glc and Xyl from a variety of different pretreatment/biomass combinations.

## Methods

### Plant materials and pretreatments

All feedstocks including corn stover were ground before pretreatment with a Wiley mill to 0.25-mm particle size instead of 0.5 mm as used earlier [4]. All biomass in the original plant samples was retained in the final ground material. For corn (*Zea mays*) stover, the AFEX conditions have been described previously [4], and this material is referred to as "GLBRC stover." The switchgrass (*Panicum virgatum*) variety was Cave-in-Rock, harvested in October 2005, and the AFEX conditions were 2:1 ammonia to biomass, 50% moisture, and 30-minute residence time at 140°C [11]. *Miscanthus *(*Miscanthus *× *gigantea*), harvested in the spring of 2005, was originally obtained from Dr. Steven Long, University of Illinois (Urbana-Champaign, IL, USA). AFEX conditions for *Miscanthus *were 2:1 ammonia to biomass loading, 233% moisture, and 5-minute residence time at 160°C [12]. Dried distillers' grains with solubles (DDGS) were a generous gift of Carbon Green Bioenergy Woodbury LLC (Lake Odessa, MI, USA). The AFEX conditions for DDGS were 1:1 ammonia to biomass loading, 60% moisture, and 15-minute residence time at 140°C [13]. Poplar (*Populus nigra*), originally obtained from the National Renewal Energy Lab (Golden, CO, USA), was treated with AFEX at 2:1 ammonia to biomass loading, 233% moisture, and 30-minute residence time at 180°C [14].

For mild base pretreatment of all feedstocks, 50 ml of 0.25% (wt/vol) NaOH (62.5 mM) was added to 1 g of feedstock in a 250-ml Erlenmeyer flask [15]. The flasks were incubated at 90°C for 4 h without shaking. The slurry was neutralized by dropwise addition of 12 N HCl, and the entire contents were dried by lyophilization before use in the enzymatic digestions. In every case, all of the plant material entering the pretreatment cycle was still present at the end of the cycle. Final glucan loadings in the enzyme digestions were adjusted to compensate for the mass of the salt introduced by the pretreatment.

	For alkaline peroxide (AP) pretreatment of all feedstocks, 50 ml of 1% H_2_O_2 _was adjusted to pH 11.5 with 5 M NaOH and mixed with 1 g of feedstock in a 250-ml Erlenmeyer flask. Final concentrations were 1% H_2_O_2 _(300 mM), 0.8% NaOH (200 mM) and 2% biomass. The flasks were incubated for 24 h at 24°C with shaking at 90 rpm. The slurries were then neutralized to pH 7 by dropwise addition of 12 N HCl. Residual H_2_O_2 _was inactivated by addition of 50 μl of catalase (28 mg protein/ml, 21,600 U/mg protein; catalog no. C30-100MG, Sigma-Aldrich, St. Louis, MO, USA). Following inactivation of the catalase by heating at 90°C for 15 minutes, the entire contents of the flasks were lyophilized before use in the enzymatic digestions. As noted above, the final glucan loadings were adjusted to compensate for the mass of the salt introduced by the pretreatment.

### Enzymes

The commercial enzyme mixtures used as benchmarks were from the same lots described in Banerjee *et al. *[5]. Multifect-Pectinase (lot no. A216353001) was a gift from Genencor, Inc. (Palo Alto, CA, USA). Individual enzymes were produced from native *Trichoderma reesei *genes expressed in *Pichia pastoris *or *T. reesei *and were from the same batches used earlier [4,5]. CBH1 (Cel7A) (from *T. longibrachiatum*), endo-β1,4-mannanase (from *Aspergillus niger*) and amyloglucosidase (γ-amylase) (from *A. niger*) were purchased from Megazyme, Ltd. (Brea, Ireland) (catalog nos. E-CBH1, E-BMANN, and E-AMGDF100, respectively).

The DOE Joint Genome Institute (JGI) identifiers for the *T. reesei *proteins used in this paper, and their alternate names and abbreviations, are CBH1 (Cel7A), Tr_123989; EG1 (Cel7B), Tr_122081; CBH2 (Cel6A), Tr_72567; Cel5A, Tr_120312; Cel12A, Tr_123232; β-glucosidase (BG), Tr_76672; endo-β1,4-xylanase 2 (EX2), Tr_123818; endo-β1,4-xylanase 3 (EX3), Tr_120229; β-xylosidase (BX), Tr_121127; Cip1, Tr_73638; Cip2 (glucuronyl esterase), Tr_123940; Cel61A, Tr_73643; α-arabinosidase 2 (Abf2), Tr_76210; and α-glucuronidase (α-Glr), Tr_72526) http://genome.jgi-psf.org/Trire2/Trire2.home.html.

### Hydrolysis

Enzyme hydrolysis was performed in a 96-deep well format using the GLBRC Enzyme Platform (GENPLAT) as described earlier [4,5]. Feedstocks were suspended and dispensed at 0.5% glucan, and final glucan loadings were 0.2%. Unless otherwise specified, enzyme loadings for all commercial benchmarks and for all mixture experiments were kept constant at 15 mg/g glucan, and reaction mixtures were incubated for 48 h at 50°C.

Design-Expert software (Stat-Ease Inc., Minneapolis, MN, USA) was used for experimental design and analysis. An augmented quadratic design was used throughout; thus, mixtures containing 6 and 16 components required 28 and 153 individual reactions, respectively. The lowest proportion of any enzyme in the core set (defined as CBH1, CBH2, EG1, BG, EX3, and BX) was set to 4%, because earlier studies indicated that for most of the core set, allowing them to go to 0% led to such poor Glc yields that reliable models could not be predicted [4]. The lowest proportion of all other enzymes ("accessory" proteins) was set to 0%. All assays were replicated once, sampled twice and assayed for Glc and Xyl twice, for a total of eight replicates of each mixture. Glc and Xyl were assayed colorimetrically [4]. Model predictions were tested experimentally as indicated in each table.

The monosaccharide composition of feedstocks was determined by the GLBRC Analytical Laboratory at Michigan State University. Briefly, samples were ground and washed sequentially with water, 70% ethanol, 1:1 chloroform:methanol, and acetone. The samples were then treated with amyloglucosidase + α-amylase, and the released Glc was quantitated as starch. The remaining material was then hydrolyzed with 2 N trifluoroacetic acid, and the released sugars were quantitated by GC of the alditol acetates. The insoluble residue from this step was treated with Updegraff's reagent, and the insoluble material was hydrolyzed with strong sulfuric acid and quantitated as cellulose using anthrone [16,17].

The proteins in the commercial preparation Novozyme 188 and in β-mannanase (Megazyme catalog E-BMANN) were analyzed using standard mass spectrometry-based proteomics [3]. Scaffold version 01_07_00 (Proteome Software, Portland, OR, USA) was used to probabilistically validate protein identifications (DOE Joint Genome Institute) using the X!Tandem and ProteinProphet computer algorithms.

## Results

### Commercial enzyme benchmarks

Two commercial *T. reesei *enzyme preparations, Spezyme CP and Accellerase 1000, were used as benchmarks. Spezyme CP contains more than 80 proteins of comparable quantity and identity to those found in the secretome of *T. reesei *RUTC30 grown on corn stover [3]. Accellerase 1000 is a *T. reesei*-based product with enhanced β-glucosidase activity http://www.genencor.com.

All combinations of three pretreatments (ammonia fiber expansion [AFEX], dilute base [0.25% NaOH] or alkaline peroxide [AP]) and five feedstocks (corn stover, switchgrass, *Miscanthus*, dried distillers' grains + solubles [DDGS] or poplar) were treated with the two commercial enzymes at loadings of 15 mg/g glucan (Table 1). Throughout this paper, Glc yields are expressed as a percentage of total Glc. Because at no stage of grinding, pretreatment, or digestion was any of the biomass discarded, the percentages reported are identical for the original feedstocks. Glc yields ranged from 17.1% to 68.6%, and Xyl yields ranged from 3.5% to 54.8% (Table 1). Accellerase 1000 was usually superior to Spezyme CP for Glc release, probably because of its enhanced β-glucosidase activity. Spezyme CP consistently released less Glc from substrates pretreated with 0.25% NaOH compared to the other pretreatments, a trend not seen with Accellerase 1000. For Xyl yields, Spezyme CP and Accellerase 1000 were approximately equal for all AFEX-pretreated biomass materials, but Spezyme CP was consistently superior for 0.25% NaOH and AP (Table 1).

**Table 1:** Commercial enzyme benchmarks for release of Glc and Xyl from 15 pretreatment/feedstock combinations*

Feedstock	Pretreatment	Glc release (%)			Xyl release (%)		
		No enzyme	Spezyme CP	Accellerase 1000	No enzyme	Spezyme CP	Accellerase 1000
Corn stover	AFEX	1.0 ± 0.0	44.0 ± 0.0	50.6 ± 0.3	1.2 ± 0.0	34.4 ± 0.5	30.0 ± 0.6
	0.25% NaOH	1.1 ± 0.0	40.7 ± 0.1	54.1 ± 0.3	1.2 ± 1.2	35.4 ± 0.2	22.9 ± 0.3
	Alk. peroxide	0.4 ± 0.0	58.4 ± 0.1	68.6 ± 0.3	0.0 ± 0.0	49.6 ± 0.6	38.0 ± 0.6
Switchgrass	AFEX	2.1 ± 0.0	22.9 ± 0.1	28.1 ± 0.0	0.0 ± 0.0	22.6 ± 0.7	23.4 ± 0.1
	0.25% NaOH	0.6 ± 0.1	16.4 ± 0.1	31.1 ± 0.1	0.0 ± 0.0	30.5 ± 0.2	23.2 ± 0.1
	Alk. peroxide	1.4 ± 0.0	41.6 ± 0.4	50.6 ± 0.1	0.0 ± 0.0	45.1 ± 0.2	34.6 ± 0.3
Miscanthus	AFEX	1.2 ± 0.3	36.2 ± 0.3	35.1 ± 0.0	0.0 ± 0.0	36.9 ± 1.0	36.8 ± 0.5
	0.25% NaOH	0.0 ± 0.0	13.0 ± 0.0	23.1 ± 0.3	0.7 ± 0.3	27.8 ± 0.2	22.2 ± 0.3
	Alk. peroxide	0.4 ± 0.1	37.7 ± 0.6	43.8 ± 0.3	0.8 ± 0.3	54.8 ± 2.2	42.3 ± 0.2
DDGS	AFEX	0.8 ± 0.1	32.6 ± 0.4	42.6 ± 0.4	1.1 ± 0.7	5.6 ± 0.2	4.9 ± 1.2
	0.25% NaOH	1.2 ± 0.1	26.4 ± 0.2	32.0 ± 0.2	0.8 ± 0.1	3.8 ± 0.8	3.5 ± 0.6
	Alk. peroxide	2.8 ± 0.1	31.5 ± 0.2	38.1 ± 0.3	2.1 ± 0.5	7.3 ± 0.6	6.4 ± 0.8
Poplar	AFEX	2.1 ± 0.3	18.0 ± 0.1	17.6 ± 0.0	0.0 ± 0.0	21.3 ± 0.8	21.5 ± 0.3
	0.25% NaOH	0.1 ± 0.1	10.3 ± 0.3	17.1 ± 0.4	0.3 ± 0.0	44.4 ± 0.1	32.3 ± 0.5
	Alk. peroxide	0.8 ± 0.0	17.8 ± 0.2	18.0 ± 0.0	0.1 ± 0.1	43.9 ± 1.4	37.3 ± 0.7

*Glc and Xyl release are expressed as a percentage of total Glc and Xyl in each substrate (Additional file 1, Table S2). Data are expressed as the means ± 1 SD (n = 8). All enzyme loadings were 15 mg/g glucan.

Comparing pretreatments, AP released more Glc than either 0.25% NaOH or AFEX from corn stover, switchgrass or *Miscanthus*, but AFEX and AP were approximately equally effective on DDGS. AP also generally outperformed the other two pretreatments for Xyl yield. None of the pretreatments with either Spezyme CP or Accellerase 1000 released Glc effectively from poplar (maximum of 18.0%), although 0.25% NaOH and AP released reasonable levels (37.3%-44.4%) of Xyl from poplar.

Comparing substrates, corn stover consistently yielded the most Glc and poplar the least. DDGS consistently yielded the least Xyl. Overall, Accellerase 1000 digestion of AP-corn stover gave the highest Glc yield (68.6%), and Spezyme CP digestion of AP-*Miscanthus *gave the highest Xyl yield (54.8%).

### Core set optimization

The relative proportions of a "core" set composed of six pure enzymes (CBH1 [Cel7A], CBH2 [Cel6A], Cel7B [EG1], a GH10 xylanase [EX3], β-glucosidase [BG], and β-xylosidase [BX]) [4,5] were independently optimized for all 15 pretreatment/substrate combinations. Total enzyme loading was held constant at 15 mg/g glucan. Table 2 shows the model prediction and experimental results for Glc, Table 3 shows the results for Xyl, and Additional file 1, Table S1 shows the optimized model and results for a 1:1 ratio of Glc and Xyl. Glc yields ranged from 9.8% (poplar pretreated with 0.25% NaOH) to 58.2% (corn stover pretreated with AP). For every substrate except poplar, for which no pretreatment gave > 13.8% yield, AP was superior to the other two pretreatments, as was also seen with the commercial enzymes (Table 1). Corn stover gave the highest yields of any substrate, followed by switchgrass.

**Table 2:** Optimized proportions of six core enzymes for release of Glc from 15 pretreatment/substrate combinations*

		Optimized enzyme proportions (%)						Glc yield (%)	
Feedstock	Pre-treatment	CBH1	BG	EG1	BX	EX3	CBH2	MP	**Exptl**.
Corn stover	AFEX	35	12	26	4	19	4	42.9	43.9 ± 0.5
	0.25% NaOH	49	5	34	4	4	4	42.0	40.7 ± 1.0
	Alk. peroxide	43	8	30	4	11	4	58.9	58.2 ± 0.2
Switchgrass	AFEX	31	4	23	4	35	4	23.0	24.4 ± 0.6
	0.25% NaOH	46	12	30	4	4	4	27.0	25.9 ± 0.8
	Alk. peroxide	47	4	19	4	22	4	38.0	39.1 ± 0.5
Miscanthus	AFEX	42	4	42	4	4	4	23.0	22.8 ± 1.3
	0.25% NaOH	36	4	46	4	5	4	17.0	17.5 ± 0.7
	Alk. peroxide	48	4	32	4	7	4	30.0	32.1 ± 1.2
DDGS	AFEX	28	20	23	4	20	4	21.3	22.6 ± 0.8
	0.25% NaOH	27	27	34	4	4	4	23.1	23.8 ± 0.5
	Alk. peroxide	16	43	29	4	4	4	31.0	29.8 ± 0.5
Poplar	AFEX	44	4	40	4	4	4	14.5	13.8 ± 0.1
	0.25% NaOH	47	4	36	4	4	4	10.0	9.8 ± 0.1
	Alk. peroxide	47	4	37	4	4	4	10.0	10.5 ± 0.2

*MP, model prediction. Exptl., experimental results. Data are expressed as means ± 1 SD (n = 8). All enzyme loadings were 15 mg/g glucan.

**Table 3:** Optimized proportions of six core enzymes for release of Xyl from 15 pretreatment/substrate combinations*

		Optimized enzyme proportions (%)						Xyl yield (%)	
		
Feedstock	Pretreatment	CBH1	BG	EG1	BX	EX3	CBH2	MP	**Exptl**.
Corn stover	AFEX	7	4	20	26	39	4	28.2	27.8 ± 0.7
	0.25% NaOH	23	4	23	23	23	4	29.0	30.1 ± 0.5
	Alk. peroxide	4	4	30	35	23	4	40.4	40.0 ± 2.0
Switchgrass	AFEX	4	4	18	23	47	4	26.2	24.8 ± 0.5
	0.25% NaOH	17	4	23	31	21	4	27.0	26.3 ± 0.6
	Alk. peroxide	4	4	4	37	47	4	40.5	39.5 ± 0.3
Miscanthus	AFEX	14	4	20	30	28	4	37.2	35.5 ± 1.0
	0.25% NaOH	20	4	28	25	19	4	22.6	23.0 ± 0.0
	Alk. peroxide	8	4	24	29	31	4	42.3	40.6 ± 0.9
DDGS	AFEX	-	-	-	-	-	-	-	< 7.0
	0.25% NaOH	-	-	-	-	-	-	-	< 7.0
	Alk. peroxide	-	-	-	-	-	-	-	< 7.0
Poplar	AFEX	4	4	11	28	47	5	19.6	21.0 ± 0.0
	0.25% NaOH	7	4	25	16	43	4	23.0	23.8 ± 1.1
	Alk. peroxide	4	4	23	19	46	4	25.2	26.0 ± 0.5

*MP, model prediction. Exptl., experimental results. Data are expressed as means ± 1 SD (n = 8). All enzyme loadings were 15 mg/g glucan. Xyl release from all DDG/pretreatment combinations was too low to construct statistically significant models.

Comparing Glc yields obtained with the commercial enzymes to the core set optimized for each pretreatment/substrate combination, the core set mixtures performed about as well as Spezyme CP for most substrate/pretreatment combinations, although yields with the core set were higher (25.9% yield vs. 16.4%) for 0.25% NaOH-switchgrass and lower for both AFEX-*Miscanthus *(22.8% vs. 36.2%) and AFEX-DDGS (22.6% vs. 32.6%). Accellerase 1000 consistently outperformed the core set for Glc yield but gave about the same yield of Xyl, consistent with our earlier results [4].

With regard to the optimal proportions of the core enzymes for Glc release from the different pretreatment/substrate combinations, CBH1 ranged from 16% (for AP-DDGS) to 49% (for 0.25% NaOH-corn stover). A noticeable trend was that the optimal proportions of EX3 were higher for AFEX-pretreated corn stover, switchgrass, and DDGS than for the other two pretreatments. For all substrates treated with 0.25% NaOH, EX3 was required only at the lowest proportion (4%-5%). This trend did not follow for *Miscanthus*, perhaps because *Miscanthus *has the lowest Xyl content of the three grass feedstocks (Additional file 1, Table S2). The elevated EX3 requirement was generally compensated by a lower loading of CBH1, i.e., CBH1 was present at a lower proportion for AFEX-pretreated corn stover, switchgrass and DDGS compared to other pretreatments. Owing to the limited importance of EX3 for releasing Glc from *Miscanthus*, CBH1 and EG1 constituted > 80% of the enzyme cocktail for this substrate, with the other enzymes needed only at their lowest limit. (Because the minimum proportion of each core enzyme was set at 4%, it cannot be determined whether a particular enzyme whose "optimum" is listed in Table 2 as 4% is needed at this level or at a lower level.) The optimum proportions of core enzymes for DDGS were different from those for corn stover, switchgrass, or *Miscanthus*, which is consistent with its very different sugar composition (Additional file 1, Table S1). For DDGS, a higher proportion of β-glucosidase and a lower proportion of CBH1 were optimal across all pretreatments (Table 2). This might be due to a lower degree of polymerization (DP) of the glucan of DDGS or to a lower degree of crystallinity.

EG1, BX, and EX3 were the most important enzymes for optimal Xyl yield, except in the case of AP-treated switchgrass, for which EG1 was needed only at the lowest limit (Table 3). The importance of EG1 in Xyl release might be attributed to the fact that EG1also hydrolyzes β1,4-xylan [4,6]. In the case of DDGS, reliable models could not be derived for any of the pretreatment conditions because Xyl yield never exceeded 7%. The low yield of Xyl might be related to the fact that DDGS has the highest percentage of Ara (6.3%) and uronic acid (3%). If these sugars are attached to xylose residues, as is typically found in cereal glucuronoarabinoxylan, then little free Xyl can be released in the absence of Abf2 and α-Glr. This hypothesis is supported by the fact that high levels of Abf2 and α-Glr are needed for optimized Xyl release from DDGS in the 16-component synthetic mixture (see next section). The statistical analyses of all models are shown in Additional file 1, Tables S5 and S6.

### Sixteen-component mixtures

As a further test of the use of GENPLAT for enzyme mixture optimization, three pretreatment/substrate combinations were studied using more complex cocktails. Since DDGS contains significant levels of starch (~5%) and Man (~2.5%) (Additional file 1, Table S2) [18], amyloglucosidase (γ-amylase) and β-mannanase were added to the cocktails. The Megazyme β-mannanase was confirmed by MS-based proteomics to be pure GenBank XP_001390707 (JGI Aspni5_50378), which is an *A. niger *protein in CAZy GH family 5 (data not shown). Three proteins that had previously been tested but found to have no effect on Glc or Xyl yield from AFEX-corn stover were also included, namely, Cip1, Cip2, and Cel12A [5]. The rationale for including them was that these proteins might be important in the presence of the other new enzymes (i.e., amyloglucosidase and β-mannanase) or on the new substrates (i.e., AP-pretreated corn stover and AFEX-pretreated DDGS). These mixtures thus contained a total of 16 proteins. All three pretreatment/substrate combinations were analyzed with all 16 enzymes.

**Figure 1:**
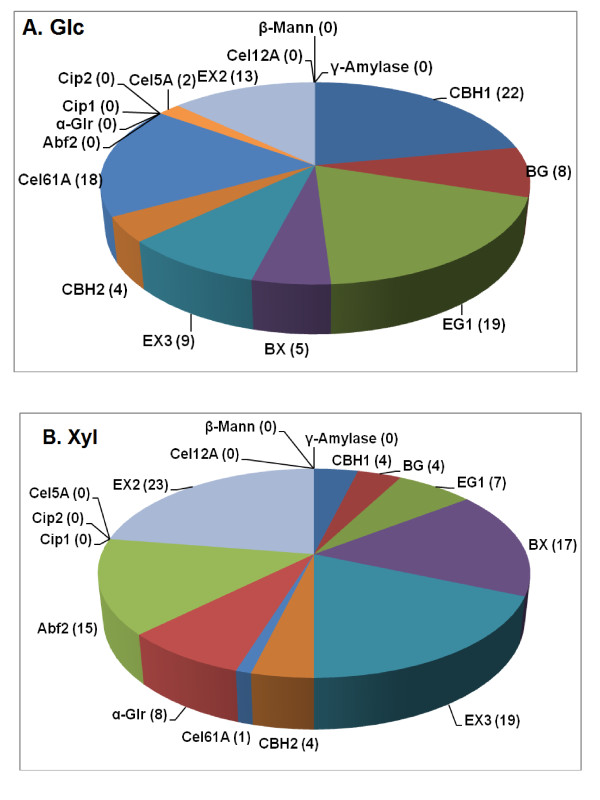
**Enzyme proportions for a 16-component synthetic mixture optimized for (a) Glc or (b) Xyl release from AFEX-corn stover**. Numbers are percentages of each enzyme in the optimized mixture. Enzyme loading was 15 mg/g glucan. Yields are shown in Figure 4. γ-Amylase is also known as amyloglucosidase.

Cip1, Cip2, and Cel12A did not contribute to Glc or Xyl release from AFEX-treated corn stover as was found earlier (Figure 1) [5]. Amyloglucosidase and β-mannanase were also unnecessary for Glc or Xyl release from this substrate, which is consistent with its low levels of starch and Man (Figure 1; Additional file 1, Table S2).

CBH1, EG1, Cel61A, and EX2 were the most important enzymes for Glc yield from AFEX-corn stover (Figure 1a), as found earlier [5], although there were some differences from the earlier results. In particular, the optimum levels of EG1 were now found to be 19%, compared to 8% in the earlier work, and the optimum level of Cel5A was 2%, compared to 11% in the earlier work. Possible reasons for this are considered in the Discussion.

**Figure 2:**
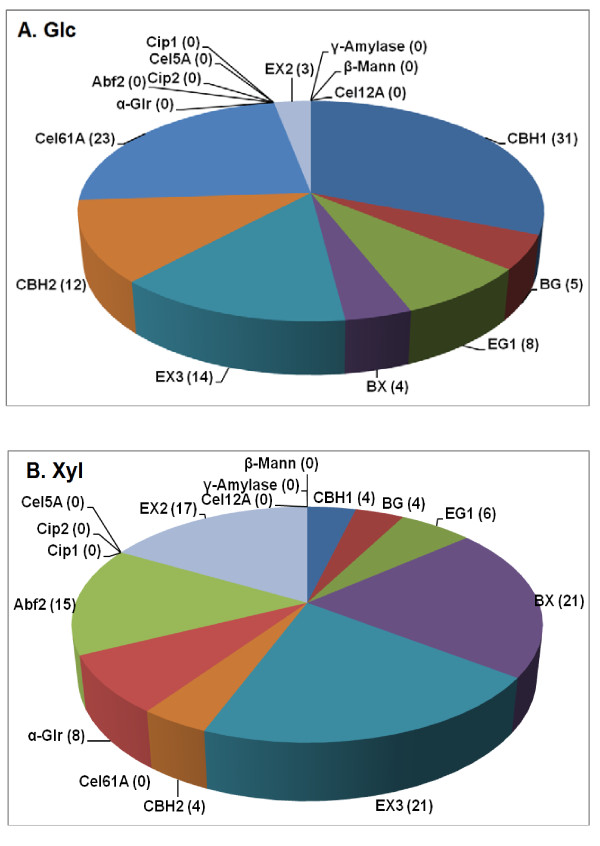
**Enzyme proportions for a 16-component mixture optimized for (a) Glc or (b) Xyl release from AP-corn stover**. Numbers are percentages of each enzyme in the optimized mixture. Enzyme loading was 15 mg/g glucan. Yields are shown in Figure 4.

Comparing AFEX- and AP-corn stover, there were some rather large differences in the optimal proportions of some of the enzymes for Glc release (Figures 1a and 2a). In particular, the optimal proportion of CBH1 for AP-corn stover was 31% compared to 22% for AFEX-corn stover, and CBH2 was also higher for AP-corn stover (12% vs. 4%). Therefore, total exo-β1,4-glucanase (CBH1 + CBH2) was 17% higher for AP-corn stover than for AFEX-corn stover. In contrast, the optimal proportions were lower for EG1 (8% vs. 19%) and Cel5A (0% vs. 2%) for AP-corn stover than for AFEX-corn stover. Therefore, total endo-β1,4-glucanase (EG1 + Cel5A) was 13% lower for AP-corn stover. Expressed in another way, the exoglucanase to endoglucanase ratio increased from 1.2:1 for AFEX-corn stover to 5.4:1 for AP-corn stover. This change in ratio indicates either that the exoglucanases are more important for AP-corn stover digestion compared to AFEX-corn stover or that the endoglucanases are less important. In the latter case, and if the exo-β1,4-glucanase activities are the limiting factor for crystalline cellulose hydrolysis, a reduced requirement for endoglucanases permits a higher proportion of the limiting factor, and this in turn could be the explanation for the enhanced Glc yields seen following AP pretreatment (Tables 1,2).

The amount of EX2 necessary for optimal Glc release decreased from 13% to 3% when comparing AP- to AFEX-corn stover (Figures 1a and 2a). Possible reasons for this are considered in the Discussion. The enzyme proportions for maximum release of Xyl from AFEX-corn stover were similar to previous results [5] and similar between AFEX- and AP-corn stover (Figures 1b and 2b).

**Figure 3:**
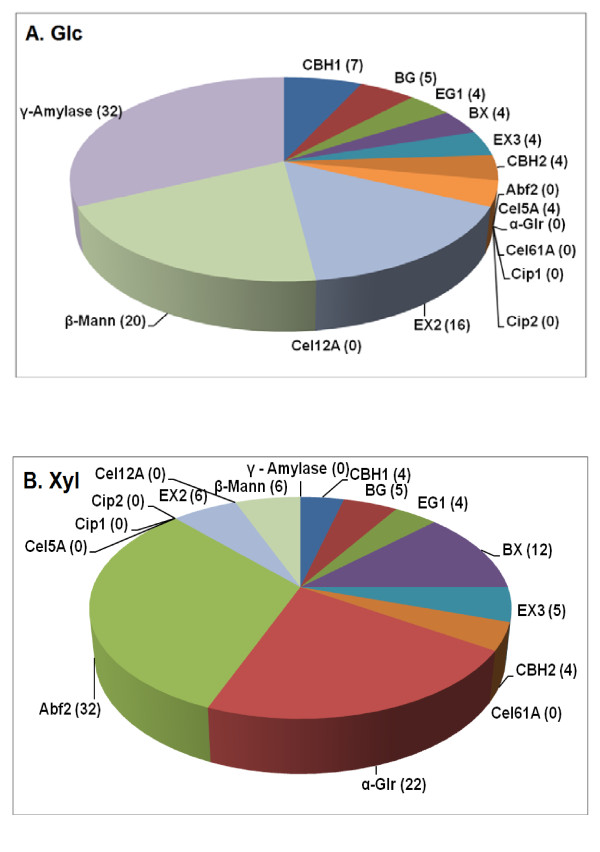
**Enzyme proportions for a 16-component mixture optimized for (a) Glc or (b) Xyl release from AFEX-DDGS**. Numbers are percentages of each enzyme in the optimized mixture. Enzyme loading was 15 mg/g glucan. Yields are shown in Figure 4.

Consistent with its different monosaccharide composition (Additional file 1, Table S2), the optimal proportions of the 16-component mixture were very different for AFEX-DDGS compared to AFEX- or AP-corn stover for release of both Glc and Xyl (Figure 3). The requirement for a high proportion of amyloglucosidase is consistent with the presence of significant residual starch in DDGS (Additional file 1, Table S2). Surprisingly, β-mannanase is the second most important enzyme for the release of Glc from DDGS (Figure 3). Possible reasons for this are considered in the Discussion section. EX2 is also necessary (at an optimum proportion of 16%), but the other enzymes are needed only at or near their lowest set levels (Figure 3). Abf2, α-Glr and BX were the most important enzymes for Xyl release from AFEX-DDGS (Figure 3b). The importance of the first two of these is consistent with the Xyl in DDGS being heavily substituted with Ara and glucuronic acid.

**Figure 4:**
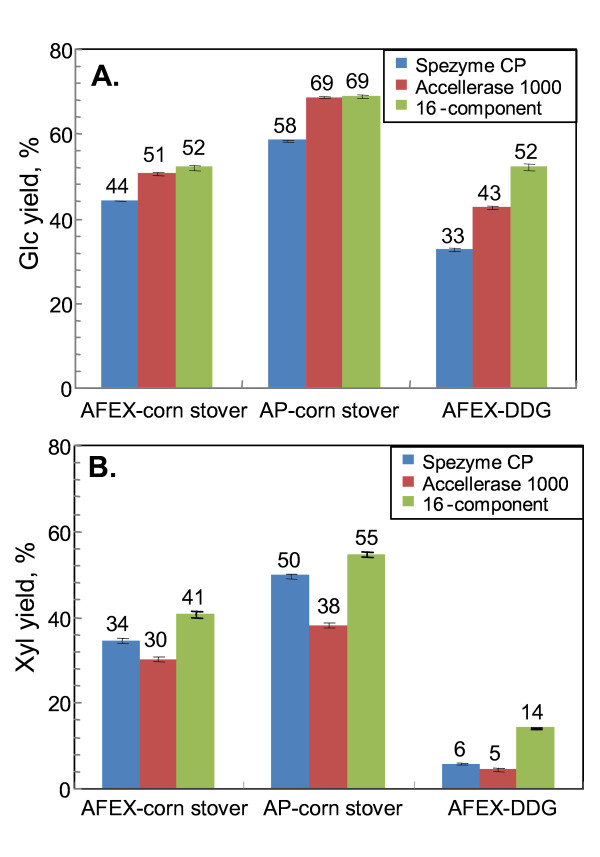
**Yields of Glc (a) and Xyl (b) for Spezyme CP, Accellerase 1000, or the 16-component optimized mixture on three pretreatment/substrate combinations**. Enzyme loadings were fixed at 15 mg/g glucan and digestions were for 48 h. Yields (as a percentage of total Glc or Xyl) are shown above the data bars. Values for Spezyme CP and Accellerase 1000 are taken from Table 1. Error bars indicate ±1 SD of the mean (n = 8).

Experimental yields of Glc and Xyl from all three pretreatment/substrate combinations with the optimized 16-component mixtures are shown in Figure 4. For ease of comparison, the yield results for Spezyme CP and Accellerase 1000 for AFEX and AP-corn stover and AFEX-DDGS from Table 1 are included in Figure 4. The 16-component mixture surpassed Spezyme CP and equaled Accellerase 1000 for Glc yield from AFEX-corn stover (Figure 4a). This result is similar to earlier results with 11-component mixtures [5], which was expected because none of the new enzymes influenced Glc yield from this substrate (Figure 1a). The 16-component mixture was also equal to Accellerase 1000 and superior to Spezyme CP for release of Glc from AP-corn stover (Figure 4a). Glc yields from AFEX-DDGS were higher with the 16-component synthetic mixture compared to either commercial preparation (Figure 4a). For Xyl, the 16-component mixture yielded more from AFEX-corn stover than either commercial preparation (Figure 4b), as previously reported [5]. This was also true for AP-corn stover (Figure 4b). Although the synthetic mixture was the best at releasing Xyl from AFEX-DDGS, it still only released 14% of the available Xyl (Figure 4b).

**Figure 5:**
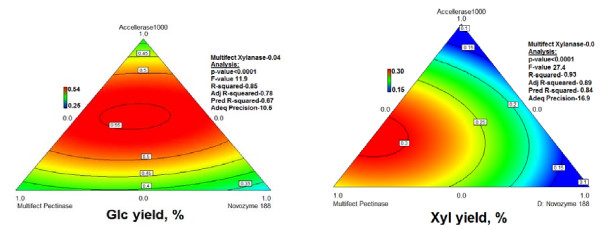
**Ternary diagrams of optimization of mixtures of four commercial enzyme preparations for release of Glc (left) or Xyl (right) from AP-treated DDG**. In these graphical representations, Multifect Xylanase was kept constant. Loadings were constant at 15 mg/g glucan. The experimental data on which the models were based are shown in Additional file 1, Table S3.

Neither Accellerase 1000 nor Spezyme CP was designed to degrade DDGS, and therefore their poor performance on this substrate is not surprising. It has been shown that mixtures of commercial enzymes can be more effective than individual ones when compared at equal protein loading (e.g., [19]). To test this, an optimization experiment was performed with four commercial enzymes on AFEX-DDGS (Figure 5). The optimized four-component mixture contained 47% Accellerase 1000, 27% Multifect Pectinase, 22% Novozyme 188 and 4% Multifect Xylanase, and outperformed all other enzyme preparations, releasing 57.2 ± 1.2% of the total Glc (Figures 4 and 5). The resulting ternary diagram is shown in Figure 5, and the underlying experimental data are shown in Additional file 1, Table S3.

Although Novozyme 188 is frequently used as a supplementary source of β-glucosidase, amyloglucosidase (glucoamylase) is actually the most abundant protein in this product, as shown experimentally by proteomics (Additional file 1, Table S4). The results in Figure 3a, which indicate that amyloglucosidase is the single most important enzyme for releasing Glc from DDGS, suggest that the superiority of the optimized four-component mixture to Accellerase 1000 alone is due, at least partially, to the high levels of amyloglucosidase present in Novozyme 188.

A mixture of the same four commercial enzymes optimized for Xyl also yielded more Xyl (29.1% ± 0.7%) from AFEX-DDGS than any of the other tested preparations (i.e., Spezyme CP, Accellerase 1000, or the 16-component synthetic mixture) (Figures 4 and 5). The mixture optimized for Xyl contained 69% Multifect Pectinase, 31% Accellerase 1000, 0% Multifect Xylanase, and 0% Novozyme 188. The paradoxical unimportance of Multifect Xylanase in this experiment might be because any necessary xylanase activity is being supplied by Multifect Pectinase [13]. The ternary diagram and experimental data for Xyl yield are shown in Figure 5 and Additional file 1, Table S3, respectively. Despite optimization, the low maximum yield of Xyl from DDGS indicates that there is still considerable scope for improvement of enzyme degradation of this substrate [13].

### Influence of loading and time on digestion of AP-corn stover

All of the preceding experiments were performed at a fixed enzyme loading (15 mg/g glucan) and a single digestion time (48 h). It is possible that synthetic enzyme mixtures show different efficiency on different substrates as a function of loading or incubation time. In particular, the higher yields seen with AP vs. AFEX-corn stover (Figure 4) led us to study the effect of loading and time on digestion of corn stover pretreated with AP.

**Figure 6:**
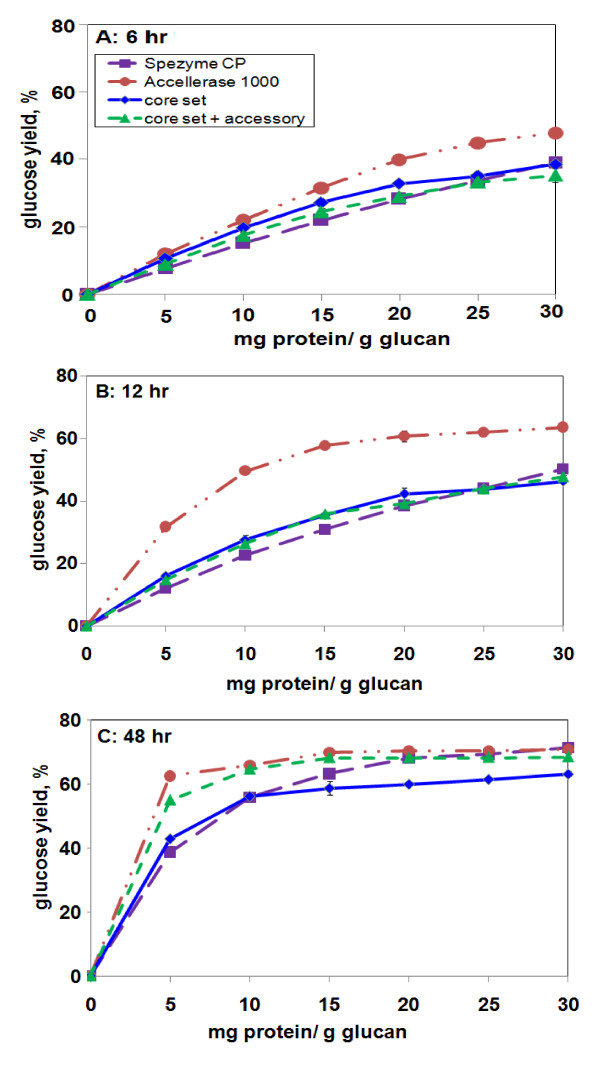
**Glc release from AP-corn stover as a function of enzyme loading**. **(a) **6 h, **(b) **12 h, **(c) **48 h. The "core set" and "core set + accessory" mixtures were identical to those used in Figures 2 and 3, i.e., optimized for 15 mg/g glucan loading and 48-h digestion. Error bars, which are sometimes obscured by the data symbols, represent ± 1 SD of the mean (n = 8).

In earlier work with AFEX-corn stover, Accellerase 1000 gave a better yield of Glc at 6 h, but Spezyme CP and an 11-component synthetic mixture were as effective as Accellerase 1000 at 48 h. At 48 h, as loading increased, Glc yields tended to plateau at ~20 mg/g glucan at a maximum yield of ~52% [5]. Because none of the extra components in the 16-component mixture used in the current paper, namely, Cip1, Cip2, Cel12A, β-mannanase, and amyloglucosidase, made any contribution to Glc yield from corn stover (Figure 2a), the 16-component results in this paper can be compared to the earlier 11-component results [5]. As shown in Figure 6, at 12 h Accellerase 1000 was superior to Spezyme CP, the core set or the 11-component mixture for Glc release from AP-corn stover. At 48 h, there was less difference between the four enzyme preparations at higher loadings. At lower loadings (< 10 mg/g glucan), Accellerase 1000 and the 16-component mixture were superior to the core set or Spezyme CP (Figure 6).

Glc release from AP-corn stover in response to Accellerase 1000, the core set, or the 16-component mixture reached a loading plateau at a lower level than Glc release from AFEX-corn stover (< 10 mg/g glucan vs. ~20 mg/g glucan) and at a higher yield (~70% vs. ~52%). This trend was especially notable for Accellerase 1000, which reached its plateau on AP-corn stover even at ~5 mg/g (Figure 6c). On AP-corn stover, therefore, Accellerase 1000 at a loading of 15 mg/g glucan released more than 60% of the available Glc in 12 h, and at a loading of 5 mg/g glucan released more than 60% of the Glc in 48 h (Figure 6). That is, under these experimental conditions, AP-pretreatment resulted in the release of more Glc in a shorter time at a lower enzyme loading than AFEX pretreatment.

**Figure 7:**
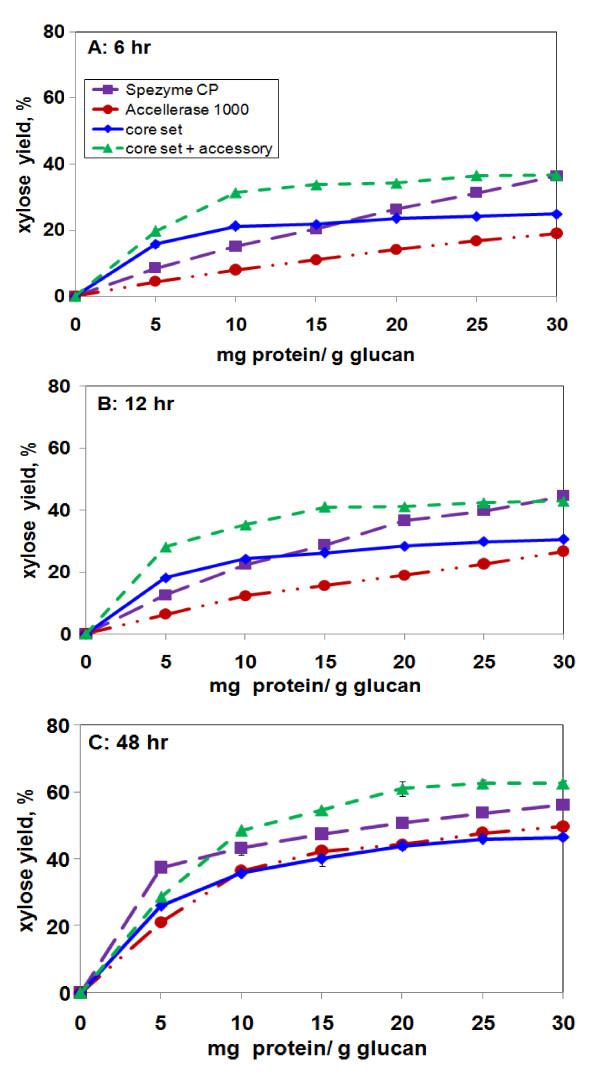
**Xyl release from AP-corn stover as a function of enzyme loading**. **(a) **6 h, **(b) **12 h, **(c) **48 h. The "core set" and "core set + accessory" mixtures were identical to those used in Figures 2 and 3, i.e., optimized for 15 mg/g glucan loading and 48-h digestion.

At 6 h, Xyl release from AP-corn stover by the two synthetic enzyme mixtures tended to reach a plateau above ~10 mg/g glucan, whereas Xyl release by the two commercial enzyme loadings was close to linear over the whole tested range of enzyme loadings (Figure 7a). This was similar to earlier results with AFEX-corn stover [5]. By 48 h, the 11-component synthetic mixture was superior to all of the others by a few percentage points (Figure 7c), which is also similar to the earlier results. For Xyl at 48 h, all of the enzymes showed a tendency to reach a plateau with increased loading, but it was not as pronounced as it was for Glc (Figures 6 and 7). As was observed for Glc yield, AP gave higher yields of Xyl than AFEX at 15 mg/g glucan and 48 h (55% vs. 41% Xyl yield with an 11-component mixture) (Figure 4b).

**Figure 8:**
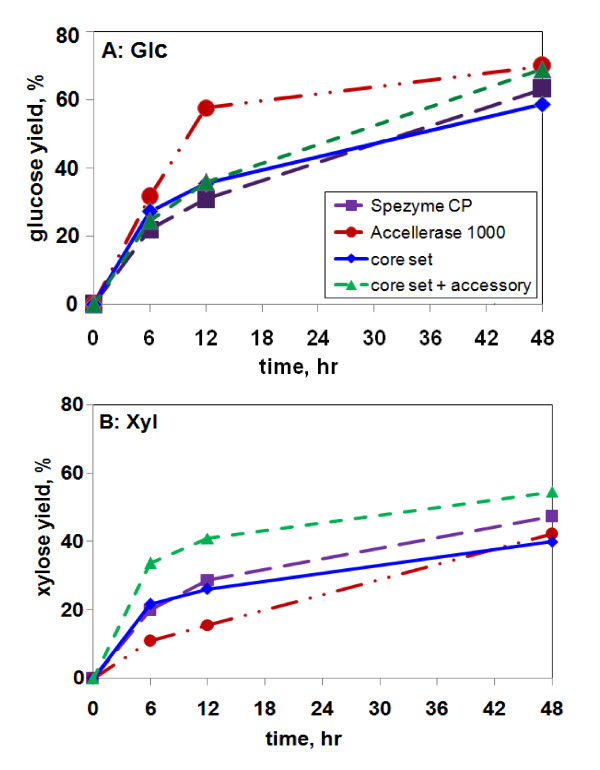
**Time course of release of Glc and Xyl from AP-corn stover**. **(a) **Glc, **(b) **Xyl. All enzyme loadings were 15 mg/g glucan. Data are from the same experiment shown in Figures 6 and 7. The "core set" and "core set + accessory" mixtures were identical to those used in Figures 2 and 3, i.e., optimized for 15 mg/g glucan loading and 48-h digestion.

The data for 15 mg/g glucan loading from Figures 6 and 7 were replotted as a function of time (Figure 8). The trends with AP-corn stover were similar to those seen with AFEX-corn stover. At 12 h, Accellerase 1000 performed the best for Glc, and the multi-component synthetic mixture performed the best for Xyl. These results suggest that the synthetic mixture still lacks one or more enzymes needed for Glc release at early time points compared to Accellerase 1000. A caveat to this is to recognize that the synthetic mixture was optimized for 48 h and might be capable of performing as well as or better than Accellerase 1000 if it were reoptimized for 12 h.

For Glc, the advantage of Accellerase 1000 disappeared at later time points as it tended to plateau more dramatically than the other enzyme mixtures (Figure 8a). For Xyl, the 16-component mixture was superior at all time points. The fact that yields of Glc and Xyl were still increasing from 12 to 48 h, in some cases almost linearly (e.g., Spezyme CP and 11-component mixture in Figure 8a and Accellerase 1000 in Figure 8B), suggests that the enzymes were still active and that the plateau effect seen at 48 h in Figures 6c and 7c was not due to enzyme inactivation.

## Discussion

The future of the lignocellulosic ethanol industry will depend on the development of economical and effective pretreatments and compatible enzyme cocktails. Many different feedstocks and pretreatments are currently being actively investigated. Insofar as regions differing in geography and climate favor the production of different feedstocks, and because different feedstocks require different pretreatments, a mature lignocellulosic ethanol industry may need to utilize a wide variety of pretreatment/feedstock combinations. Since it is unlikely that only one or a few enzyme cocktails will be sufficiently effective in all cases, it will be necessary to develop custom cocktails. GENPLAT (or a scaled-up version of it) should prove to be useful for the development of such custom cocktails.

In this paper, we show the use of GENPLAT to rapidly determine the optimal proportions of enzymes in complex synthetic mixtures for the digestion of a variety of pretreated biomass feedstocks. Most of these results are consistent with other studies and with our earlier results, e.g., the importance of core enzymes such as CBH1, EG1, and BG for Glc release; the importance of EX, BX, Abf2, and α-Glr for Xyl release; the importance of EX for Glc release; the importance of the cryptic endoglucanase known as Cel61 for Glc release; and the involvement of amyloglucosidase for Glc release from DDGS [4,5]. However, other results reported here were unexpected from previous studies.

First, we found that the optimal proportions of several enzymes were significantly different in the current experiments compared with those we reported earlier [5]. In particular, in the 16-component experiments (which are comparable to the earlier 11-component experiments [5]), the new optimized model predicted a large shift in the optimal proportions of EG1 (i.e., an increase from 8% to 19%) and of Cel5A (i.e., a decrease from 11% to 2%). A possible explanation for this observation might lie in the fact that EG1 and Cel5A are both endo-β1,4-glucanases and therefore have overlapping, or even identical, functions in the degradation of lignocellulose. Large changes in the relative proportions of these two, while maintaining their sum constant (i.e., the sum of EG1 + Cel5A was 19% in the earlier experiments and 21% in the current ones) might result in a very small shift in Glc yield, resulting in different but equally valid models. That is, the topology of the response surface in the EG1/Cel5A region might be relatively flat, and minor experimental variations could change their relative proportions greatly without having a major effect on Glc yield. If these two enzymes are essentially interchangeable, the choice of which one to use in synthetic mixtures might not make much difference and could be dictated by other factors such as cost.

Another example of such "equivalence switching" might occur in the 16-component experiments comparing AFEX-corn stover and AP-corn stover (Figures 1 and 2). As found earlier [5], for both substrates a combination of two endo-β1,4-xylanases (EX2 and EX3) is superior to either one alone (Figures 1 and 2). The reason for this is not clear, but it indicates that the functions of the two enzymes are not completely overlapping. In the current experiments, shown in Figures 1 and 2, AP-corn stover requires more EX3 (14% vs. 9%) but less EX2 (3% vs. 13%). The sum of EX2 + EX3, however, changes only from 22% to 17%. Compared to the two endo-β1,4-glucanases, however, in this case the situation is complicated by the fact that the two endoxylanases are not catalytically equivalent. EX2 (GH family 11) is specific for xylan, whereas EX3 (GH 10) has a broader range of substrates, including β1,4-glucan oligomers [20].

In contrast to EG1 and Cel5A, the optimum proportions of Cel61A for Glc release stayed fairly constant throughout the experiments. Optimum proportions were 17% (AFEX-corn stover) [5], 18% (AFEX-corn stover; Figure 1a) and 23% (AP-corn stover; Figure 2a). Cel61A was not needed for Xyl release from corn stover nor for either Glc or Xyl release from AFEX-DDGS (Figure 3). Although the catalytic function of Cel61A is still uncertain, especially on complex polysaccharides, we have found that it can hydrolyze cellopentaose to cellobiose and cellotriose, and cellohexaose to cellobiose (unpublished observations). Nonetheless, our experiments indicate that Cel61A is not redundant with other endo-β1,4-glucanases, which can also act on these same cello-oligosaccharides, and that it therefore probably plays some other, unknown role in cell wall degradation [21]. Our empirical determination of the importance of Cel61A illustrates that it can be difficult to predict which enzymes will be important, and at what proportions, based solely on our (imperfect) knowledge of the structures of plant cell walls and our (imperfect) knowledge of the full range of enzymatic activities of the large number of proteins secreted by lignocellulolytic microorganisms [3].

Another unexpected result was the need for a high level of β-mannanase to release Glc from AFEX-DDGS (Figure 3a). Although DDGS contains only 2.5% Man, polymers of Man apparently play an important role in the cell wall structure of DDGS. Hägglund *et al. *[22] found that the CBM module of fungal β-mannanase binds to cellulose and promotes the hydrolysis of cellulose in mannan/cellulose complexes, but plays no role in the hydrolysis of pure cellulose. There is evidence that mannan in plant cell walls is tightly bound to cellulose microfibrils [23,24]. From these observations, a plausible explanation for the importance of β-mannanase is that in DDGS, and perhaps some other lignocellulosic materials, polymers of Man must be hydrolyzed before the true cellulases can access cellulose. It is difficult to speculate further about the importance of β-mannanase in the degradation of DDGS because little is known about the cell wall structure of corn DDGS. Most analyses of the "glucan" in DDGS have not discriminated between the different polymers in which Glc could be present. These include not only crystalline cellulose and starch but also amorphous cellulose, mixed-linked glucan, and glucomannan. For example, Kim *et al. *[25] assumed that all nonstarch Glc in DDGS is derived from "cellulose," but it is known that the endosperm tissues of barley and other cereals can contain high levels of Glc in the form of mixed-linked glucan [26].

Another paradox about DDGS is our observation that the core set of enzymes (which includes EX3) was much less effective at releasing Xyl from DDGS than from AFEX-corn stover [4]. It is also surprising that the exo-β1,4-glucanases and the endo-β1,4-glucanases were relatively unnecessary for releasing Glc from DDGS compared to the stovers (Figure 3a), even though total Glc yields from DDGS were comparable to those of AFEX-corn stover (Figure 4a). Collectively, our enzyme optimization results suggest that our knowledge of the structures and interactions of polysasccharides in DDGS is still very imperfect.

The importance of xylanases (in the case of grass stovers) and β-mannanase (in the case of DDGS) for Glc yield are consistent with hemicelluloses in the cell wall having a major effect on limiting access of cellulases to cellulose. Collectively, enzymes that degrade the hemicelluloses thus play two roles in industrial lignocellulose degradation: promotion of access of cellulases to cellulose and release of fermentable hemicellulosic monosaccharides.

## Conclusions

Improvement in the efficiency of enzyme mixtures, either mixtures of commercial enzymes or synthetic mixtures of pure enzymes, would greatly improve the economic viability of lignocellulosic ethanol. Results from many laboratories have contributed to the identification of the critical enzymes needed for release of Glc and Xyl from pretreated biomass. However, we predict that there are additional critical enzymes that are either absent or present at suboptimal levels in commercial enzyme preparations. GENPLAT provides a technology by which such enzymes can be identified and validated.

Our results also illustrate the difficulty of predicting which enzymes are necessary to degrade a particular pretreatment/biomass substrate based on either monosaccharide or polysaccharide abundance. Therefore, the development of better enzyme cocktails will require empirical approaches.

## Abbreviations

Abf2: arabinosidase 2; AFEX: ammonia fiber expansion; α-Glr: α-glucuronidase; AP: alkaline peroxide; Ara: arabinose; BG: β-glucosidase; BX: β-xylosidase; CBH: cellobiohydrolase; DDGS: dried distillers' grains plus solubles; EG: endo-β1,4-glucanase; EX: endo-β1,4-xylanase; Glc: glucose; Man: mannose; Xyl: xylose.

## Competing interests

The authors declare that they have no competing interests.

## Authors' contributions

GB and SC performed the alkaline peroxide and dilute base pretreatments, expressed some of the proteins, designed and executed the digestion experiments, performed the statistical analyses and helped write the paper; JSC and MB cloned genes and expressed proteins and helped write the paper; JDW contributed to experimental design and drafted the final manuscript. All authors provided input to the manuscript and read and approved the final manuscript.

## Supplementary Material

Additional file 1 **Supplementary supporting data**. Supplementary Table S1. Optimized proportions of the core set for a 1:1 yield of Glc and Xyl. Supplementary Table S2. Monosaccharide and lignin composition of feedstocks used in this paper. Supplementary Table S3. Experimental results for optimization of digestion of AP-treated DDGS with mixtures of four commercial enzyme preparations. Supplementary Table S4. Proteomic analysis of the commercial enzyme product Novozyme 188. Supplementary Table S5. Statistical analysis for Glc optimization from pretreatment/substrate combinations. Supplementary Table S6. Statistical analysis for Xyl optimization from pretreatment/substrate combinations. Click here for file
