# Supplementary material for: Rapid optimization of enzyme mixtures for deconstruction of diverse pretreatment/biomass feedstock combinations
Source: Biotechnol Biofuels. 2010 Oct 12;3:22. doi: 10.1186/1754-6834-3-22 (PMC2964541; doi:10.1186/1754-6834-3-22)
Supplement: Additional file 1 — Supplementary supporting data. Supplementary Table S1. Optimized proportions of the core set for a 1:1 yield of Glc and Xyl. Supplementary Table S2. Monosaccharide and lignin composition of feedstocks used in this paper. Supplementary Table S3. Experimental results for optimization of digestion of AP-treated DDGS with mixtures of four commercial enzyme preparations. Supplementary Table S4. Proteomic analysis of the commercial enzyme product Novozyme 188. Supplementary Table S5. Statistical analysis for Glc optimization from pretreatment/substrate combinations. Supplementary Table S6. Statistical analysis for Xyl optimization from pretreatment/substrate combinations. [file 1754-6834-3-22-S1.DOC]

September 17, 2010

**Supplementary Tables for Banerjee et al. “Rapid optimization of enzyme mixtures for deconstruction of diverse pretreatment/biomass feedstock combinations”**

**Supplementary Table S1** Optimized proportions of the core set for a 1:1 yield of Glc and Xyl. MP, model prediction. Exptl., experimental results with the model prediction proportions.

| Feedstock | Pre-treatment | Optimized enzyme proportions ( %) | | | | | | Glc yield (%) | | Xyl yield (%) | |
| --- | --- | --- | --- | --- | --- | --- | --- | --- | --- | --- | --- |
|  |  | CBH1 | BG | EG1 | BX | EX3 | CBH2 | MP | Exptl. | MP | Exptl. |
| corn stover | AFEX | 30 | 4 | 29 | 4 | 29 | 4 | 41.7 | 42.0 ± 0.5 | 27.9 | 28.0 ± 1.1 |
|  | 0.25% NaOH | 46 | 4 | 38 | 4 | 4 | 4 | 41.7 | 41.2 ± 0.3 | 25.2 | 27.0 ± 0.5 |
|  | Alk. peroxide | 41 | 4 | 23 | 4 | 24 | 4 | 58.5 | 58.5 ± 0.9 | 36.9 | 35.3 ± 1.5 |
| switchgrass | AFEX | 28 | 4 | 20 | 4 | 40 | 4 | 22.9 | 24.2 ± 1.0 | 25.7 | 27.0 ± 0.5 |
|  | 0.25% NaOH | 40 | 4 | 24 | 4 | 24 | 4 | 25.6 | 26.0 ± 0.5 | 25.1 | 26.2 ± 0.1 |
|  | Alk. peroxide | 45 | 4 | 4 | 4 | 39 | 4 | 36.7 | 36.8 ± 0.5 | 38.2 | 37.0 ± 0.5 |
| Miscanthus | AFEX | 39 | 4 | 41 | 4 | 8 | 4 | 23.3 | 25.0 ± 1.2 | 30.1 | 32.0 ± 1.4 |
|  | 0.25% NaOH | 34 | 4 | 34 | 4 | 20 | 4 | 16.5 | 18.8 ± 1.3 | 21.6 | 21.0 ± 1.0 |
|  | Alk. peroxide | 37 | 4 | 28 | 4 | 23 | 4 | 28.9 | 26.0 ± 0.5 | 39.9 | 42.0 ± 2.1 |
| DDGS | AFEX | n.a. | n.a. | n.a. | n.a. | n.a. | n.a. | n.a. | n.a. | n.a. | n.a. |
|  | 0.25% NaOH | n.a. | n.a. | n.a. | n.a. | n.a. | n.a. | n.a. | n.a. | n.a. | n.a. |
|  | Alk. peroxide | n.a. | n.a. | n.a. | n.a. | n.a. | n.a. | n.a. | n.a. | n.a. | n.a. |
| poplar | AFEX | 37 | 4 | 29 | 4 | 21 | 5 | 13.4 | 14.0 ± 0.2 | 18.4 | 18.0 ± 0.2 |
|  | 0.25% NaOH | 42 | 4 | 28 | 4 | 18 | 4 | 8.5 | 10.0 ± 0.5 | 20.6 | 22.0 ± 0.5 |
|  | Alk. peroxide | 25 | 4 | 26 | 4 | 37 | 4 | 8.5 | 9.50 ± 0.2 | 24.8 | 22.9 ± 1.3 |

**Supplementary Table S2**  Monosaccharide and lignin composition of feedstocks used in this paper. Analyses were done prior to pretreatments. Data are % of dry weight. N.D., not determined.

| Feedstock | Glc | | Xyl | Ara | Man | Gal | Uronic acid | Lignin | total |
| --- | --- | --- | --- | --- | --- | --- | --- | --- | --- |
|  | total Glc | starch |  |  |  |  |  |  |  |
| corn stover | 34.4 | <1% | 22.4 | 4.2 | 0.6 | 1.4 | 2.0 | 11.0 | 76 |
| switchgrass | 35 | N.D. | 25.3 | 2.0 | 0.3 | 1.0 | 2.0 | 16.7 | 82.3 |
| Miscanthus | 44 | N.D. | 19.0 | 1.8 | 0.1 | 0.4 | 1.8 | 24.1 | 90.9 |
| DDGS | 22 | 5.0 | 8.0 | 6.3 | 2.5 | 3.0 | 3.0 | < 5 | 49.8 |
| poplar | 43.8 | N.D. | 14.9 | 0.6 | 3.9 | 1.0 | N.D. | 29.1 | 93.3 |

**Supplementary Table S3**  Experimental results for optimization of digestion of AP-treated DDGS with mixtures of four commercial enzyme preparations. Loading was fixed at 15 mg protein/g glucan.

| **Enzyme Proportion** | | | | **% Glc yield** | **% Xyl yield** |
| --- | --- | --- | --- | --- | --- |
| Accellerase 1000 | Multifect- Xylanase | Multifect-Pectinase | Novozyme 188 |
| 1.00 | 0.00 | 0.00 | 0.00 | 38.5 ± 0.0 | 8.6 ± 0.8 |
| 0.00 | 1.00 | 0.00 | 0.00 | 24.6 ± 0.0 | 17.7 ± 0.6 |
| 0.00 | 0.00 | 1.00 | 0.00 | 34.6 ± 0.1 | 26.5 ± 0.9 |
| 0.00 | 0.00 | 0.00 | 1.00 | 28.7 ± 0.0 | 8.1 ± 0.1 |
| 0.50 | 0.50 | 0.00 | 0.00 | 40.2 ± 0.0 | 17.6 ± 2.3 |
| 0.50 | 0.00 | 0.50 | 0.00 | 51.7 ± 0.1 | 28.5 ± 0.0 |
| 0.50 | 0.00 | 0.00 | 0.50 | 50.9 ± 0.2 | 16.5 ± 2.1 |
| 0.00 | 0.50 | 0.50 | 0.00 | 40.7 ± 0.1 | 26.0 ± 0.7 |
| 0.00 | 0.50 | 0.00 | 0.50 | 35.8 ± 0.0 | 15.3 ± 0.4 |
| 0.00 | 0.00 | 0.50 | 0.50 | 34.1 ± 0.1 | 22.6 ± 0.5 |
| 0.63 | 0.13 | 0.13 | 0.13 | 51.9 ± 0.2 | 21.3 ± 0.6 |
| 0.13 | 0.63 | 0.13 | 0.13 | 51.7 ± 0.1 | 23.7 ± 0.4 |
| 0.13 | 0.13 | 0.63 | 0.13 | 53.2 ± 0.3 | 29.4 ± 1.4 |
| 0.13 | 0.13 | 0.13 | 0.63 | 51.7 ± 0.2 | 24.5 ± 0.4 |
| 0.25 | 0.25 | 0.25 | 0.25 | 52.9 ± 0.2 | 25.9 ± 1.1 |

**Supplementary Table S4** Proteomics analysis of the commercial enzyme product Novozyme 188. The JGI Protein ID’s are from the Department of Energy Joint Genome Institute (http://genome.jgi-psf.org/Aspni5/Aspni5.home.html). All hits with more than two peptides and a probability >95% as calculated by Scaffold are shown. No proteins from species other than *Aspergillus* *niger* were identified.

| JGI Protein ID | Annotation | Spectral counts |
| --- | --- | --- |
| 213597 | Amyloglucosidase (Glucoamylase) (GH15) | 448 |
| 56782 | β-glucosidase (GH3) | 90 |
| 47911 | α-amylase | 31 |
| 211032 | Hypothetical tripeptidyl peptidase | 21 |
| 56553 | Hypothetical cell wall protein | 20 |
| 201655 | Aspergillopepsin A-like | 15 |
| 205361 | Hypothetical protein | 12 |
| 44517 | Cell wall glucanase (GH17) | 11 |
| 50599 | Hypothetical protein | 10 |
| 35378 | Hypothetical protein | 8 |
| 54398 | β-N-acetylhexosaminidase (GH20) | 8 |
| 46979 | Carboxypeptidase CpdS | 8 |
| 214786 | Hypothetical protein | 7 |
| 52703 | Hypothetical protein | 7 |
| 55270 | Exo-β-1,3-glucanase | 6 |
| 57436 | Endo-β-1,4-xylanase | 6 |
| 196122 | Cell wall glucanase Crf1 (GH16) | 6 |
| 53033 | 1,3-β-glucanosyltransferase | 5 |
| 37736 | α-galactosidase A | 3 |
| 214608 | Endoglucanse A (GH5) | 2 |
| 54865 | Hypothetical protein | 2 |

**Supplementary Table S5** Statistical analysis for Glc optimization from pretreatment/substrate combinations (see Tables 2, 3, and Figures 1, 2, 3).

| Feedstock | Pre-treatment | No. of components | p-value | F-value | R^2 | Adjusted R^2 | Predicted R^2 | Difference between Adj and Pred R^2 | Adequate Precision |
| --- | --- | --- | --- | --- | --- | --- | --- | --- | --- |
| corn stover | AFEX | 6 | <0.0001 | 21.6 | 0.93 | 0.88 | 0.84 | 0.04 | 17.8 |
|  |  | 16 | <0.0001 | 12.4 | 0.60 | 0.55 | 0.43 | 0.12 | 17.6 |
|  | Mild Base | 6 | <0.0001 | 15.6 | 0.90 | 0.84 | 0.76 | 0.08 | 14.5 |
|  | Alk. peroxide | 6 | <0.0001 | 13.6 | 0.89 | 0.82 | 0.74 | 0.08 | 14.5 |
|  |  | 16 | <0.0001 | 13.9 | 0.60 | 0.55 | 0.43 | 0.12 | 19.4 |
| switchgrass | AFEX | 6 | <0.0001 | 13.0 | 0.88 | 0.82 | 0.80 | 0.02 | 14.3 |
|  | Mild Base | 6 | <0.0001 | 11.6 | 0.87 | 0.80 | 0.70 | 0.10 | 12.3 |
|  | Alk. peroxide | 6 | <0.0001 | 11.0 | 0.86 | 0.78 | 0.72 | 0.06 | 12.2 |
| Miscanthus | AFEX | 6 | <0.0001 | 11.7 | 0.87 | 0.80 | 0.70 | 0.10 | 13.9 |
|  | Mild Base | 6 | <0.0001 | 9.0 | 0.84 | 0.74 | 0.70 | 0.04 | 11.1 |
|  | Alk. peroxide | 6 | <0.0001 | 8.2 | 0.83 | 0.73 | 0.60 | 0.13 | 11.1 |
| DDGS | AFEX | 6 | <0.0001 | 14.0 | 0.90 | 0.83 | 0.75 | 0.08 | 15.0 |
|  |  | 16 | <0.0001 | 16.8 | 0.66 | 0.62 | 0.52 | 0.10 | 18.8 |
|  | Mild Base | 6 | <0.0001 | 19.0 | 0.92 | 0.87 | 0.82 | 0.05 | 14.7 |
|  | Alk. peroxide | 6 | <0.0001 | 11.6 | 0.87 | 0.80 | 0.67 | 0.13 | 15.1 |
| poplar | AFEX | 6 | <0.0001 | 15.8 | 0.90 | 0.85 | 0.76 | 0.09 | 16.7 |
|  | Mild Base | 6 | <0.0001 | 6.0 | 0.77 | 0.63 | 0.60 | 0.03 | 9.2 |
|  | Alk. peroxide | 6 | <0.0001 | 22.6 | 0.77 | 0.73 | 0.72 | 0.01 | 17.4 |

**Supplementary Table S6** Statistical analysis for Xyl optimization from pretreatment/substrate combinations (see Tables 2, 3, and Figures 1, 2, 3).

| Feedstock | Pre-treatment | No. of components | p-value | F-value | R^2 | Adjusted R^2 | Predicted R^2 | Difference between Adj and Pred R^2 | Adequate Precision |
| --- | --- | --- | --- | --- | --- | --- | --- | --- | --- |
| corn stover | AFEX | 6 | <0.0001 | 17.0 | 0.91 | 0.86 | 0.75 | 0.11 | 15.5 |
|  |  | 16 | <0.0001 | 9.9 | 0.55 | 0.50 | 0.45 | 0.05 | 14.5 |
|  | Mild Base | 6 | <0.0001 | 26.3 | 0.94 | 0.90 | 0.88 | 0.02 | 24.9 |
|  | Alk. peroxide | 6 | <0.0001 | 16.2 | 0.90 | 0.85 | 0.78 | 0.07 | 16.5 |
|  |  | 16 | <0.0001 | 10.0 | 0.51 | 0.46 | 0.31 | 0.15 | 14.5 |
| switchgrass | AFEX | 6 | <0.0001 | 17.1 | 0.91 | 0.86 | 0.81 | 0.05 | 14.5 |
|  | Mild Base | 6 | <0.0001 | 9.5 | 0.85 | 0.76 | 0.61 | 0.15 | 11.6 |
|  | Alk. peroxide | 6 | <0.0001 | 19.7 | 0.92 | 0.87 | 0.83 | 0.04 | 17.6 |
| Miscanthus | AFEX | 6 | <0.0001 | 13.0 | 0.88 | 0.82 | 0.74 | 0.08 | 15.4 |
|  | Mild Base | 6 | <0.0001 | 7.2 | 0.81 | 0.70 | 0.68 | 0.02 | 10.3 |
|  | Alk. peroxide | 6 | <0.0001 | 9.7 | 0.85 | 0.76 | 0.66 | 0.10 | 12.0 |
| DDGS | AFEX | 6 | n.d. | n.d. | n.d. | n.d. | n.d. | n.d. | n.d. |
|  |  | 16 | <0.0001 | 10.7 | 0.57 | 0.52 | 0.37 | 0.15 | 21.7 |
|  | Mild Base | 6 | n.d. | n.d. | n.d. | n.d. | n.d. | n.d. | n.d. |
|  | Alk. peroxide | 6 | n.d. | n.d. | n.d. | n.d. | n.d. | n.d. | n.d. |
| poplar | AFEX | 6 | <0.0001 | 7.6 | 0.82 | 0.71 | 0.57 | 0.14 | 11.0 |
|  | Mild Base | 6 | <0.0001 | 32.3 | 0.95 | 0.92 | 0.88 | 0.04 | 18.7 |
|  | Alk. peroxide | 6 | <0.0001 | 28.4 | 0.94 | 0.91 | 0.87 | 0.04 | 19.4 |
